# Adult-Onset Myoclonus: Comparisons and Contrasts in the Inpatient and Outpatient Settings

**DOI:** 10.5334/tohm.1077

**Published:** 2025-11-10

**Authors:** Moath Hamed, Karin Oh, Donna Zarandi, Moyosore Oluleye, Anas Zaher, Jude Elsaygh, Shaheen Rizly, Xiaoyue Ma, Hwai Yin Ooi, Harini Sarva, Miran Salgado, Daryl Victor

**Affiliations:** 1 Department of Neurology, New York Presbyterian Brooklyn Methodist Hospital, Brooklyn, NY, USA; 2 Department of Neurology, Weill Cornell Medicine, New York, NY, USA; 3 Department of Neurology, Columbia University Irving Medical Center, New York, NY, USA; 4 Department of Internal Medicine, New York Presbyterian Brooklyn Methodist Hospital, Brooklyn, NY, USA; 5 Department of Population Health Sciences, Weill Cornell Medicine, New York, NY, USA

**Keywords:** myoclonus, asterixis

## Abstract

**Background:**

Myoclonus is a hyperkinetic movement disorder presenting as rapid jerky involuntary movements. The etiology of myoclonus differs between in-hospital and outpatient clinic settings. Historically, those in the hospital typically develop myoclonus from toxic-metabolic or hypoxic-ischemic etiologies, whereas those presenting to a clinic tend to have an underlying neurodegenerative etiology.

**Methods:**

We retrospectively reviewed charts of both inpatient and outpatient adult cases with myoclonus at New York Presbyterian Brooklyn Methodist Hospital over 10 years. Data were analyzed with descriptive statistical methods to elucidate demographics, etiologies, and outcomes.

**Results:**

Overall, 279 inpatient (56.63% female aged 70.61 + 15.76 years) and 85 outpatient (52.9% female aged 64.3 + 16.3 years) individuals were included in our study. Outpatient cases were younger on average than inpatient counterparts (*p* < 0.05). While more Caucasian individuals were seen in the outpatient setting, more black individuals were seen in the inpatient setting; ethnic distributions did not differ significantly between the two cohorts (*p* > 0.05). Longer symptom duration was prevalent in outpatient cases (IQR 3–45 months) compared to inpatient (IQR < 1–4 days) ones (*p* < 0.05). Etiological distributions varied between the two cohorts, with toxic/drug-induced, metabolic (non-genetic), and static hypoxic/ischemic etiologies predominating our inpatient cohort, and neurodegenerative, inflammatory/autoimmune/paraneoplastic, and idiopathic etiologies more prevalent in the outpatient setting. Spinal nervous system lesion and toxic/drug-induced outpatient cases tended to present focally, but inflammatory/autoimmune/paraneoplastic etiologies were associated with axial-predominant symptoms among our outpatient cohort (*p* < 0.05). Responses to treatment of underlying etiology and/or anti-seizure drugs was robust in both settings overall, with over 70% of individual cases showing response.

**Conclusions:**

Myoclonus in the inpatient and outpatient settings have differences in etiology and symptom duration, with longer duration and more neurodegenerative and inflammatory/autoimmune/paraneoplastic etiologies predominating in the outpatient cohort compared to the inpatient one. List of causes of myoclonus do not typically differentiate between the presentation in inpatient and outpatient settings. If the causes differ by setting, listing causes by setting may aid clinicians in ranking *a priori* probabilities.

Myoclonus is a hyperkinetic movement disorder consisting of brief, shock-like involuntary movements due to muscular contractions (so-called “positive” myoclonus) or inhibitions (or “negative” myoclonus (asterixis)) [1]. Classification of myoclonus is achieved through identifying several axes in accordance with the latest IAPRD consensus definition: localization (cortical, cortical-subcortical, subcortical, brainstem, peripheral, spinal, propriospinal, and functional); and etiology (genetic, acquired, functional, idiopathic, and physiological) [1,2,3]. Acquired cases consist of toxic/drug-induced (iatrogenic), metabolic (non-genetic), neurodegenerative, lesional (static or progressive), infectious/post-infectious, inflammatory/autoimmune/paraneoplastic, and other medical or systemic disorders [1,2,3]. Classification is attained through careful history-taking, comprehensive medication review, detailed neurological examination, routine laboratory testing, dedicated neuroaxis imaging, and electrophysiological testing, with subsequent etiology-specific or symptomatic treatments available to treat the underlying condition and/or the myoclonus itself [4,5]. Differential diagnosis for myoclonus includes tics, dystonia, chorea, stereotypies, and tremor (Table 1) [4]. Careful observation of the tempo (onset, duration, and termination) of movements, the presence or absence of a reflex or trigger, its suppressibility, pattern, and the results of neurophysiological testing (electroencephalography and/or electromyography) help distinguish myoclonus from other hyperkinetic movement disorders [6,7,8].

**Table 1:** Distinguishing features of myoclonus and other movement disorders.


	MYOCLONUS	TICS	DYSTONIA	TREMOR	CHOREA

Duration/Cadence	Very brief, shock-like	Brief	Sustained, longer duration	Sustained, longer duration	Could be brief

Onset	Abrupt	Abrupt	Gradual	Gradual	Rapid

Reflex	Frequent	Premonitory urge	May be kinesigenic	Rest, postural, or action	No

Termination	Abrupt	Abrupt	Progressive	Progressive	Progressive

Suppressibility	No	Temporary	No	Temporary	No

Pattern	Simple	Simple/complex	Multiplanar complex	Sinusoidal, rhythmic	Flows from one body part to another

Neurophysiological testing	Back-averaging EEG potentials preceding EMG	Organization of the movement	May be useful for treatment	EMG/NCS can distinguish action tremor from myoclonus	Unhelpful


While cases of myoclonus abound in scientific literature, classifying and diagnosing myoclonus in the clinical setting remains challenging, and many hyperkinetic movements can be misclassified as myoclonus [9]. At the same time, however, myoclonus subtypes are difficult to distinguish from one another based on semiological characteristics alone, and further electrodiagnostic testing may be required to elucidate these subtypes [9]. A study of post-anoxic myoclonus in the Netherlands found that clinical assessment alone is highly variable amongst different physicians [10]. Another study of 66 individuals in the Netherlands found an improvement in inter-rater agreement of myoclonus but still poor-to-moderate inter-rater agreement with localizing or subtyping it [11]. Differentiating cortical myoclonus from essential, parkinsonian, and dystonic tremor can be equally challenging, with another group in the Netherlands finding that 37% of 773 cases had changes in their diagnosis following electromyographic and accelerometric testing [12]. Our group recently published a study looking at adult-onset myoclonus in the inpatient setting and found that over half (approximately 56%) of cases of myoclonus in our study were not initially identified as such by the admitting medical team, suggesting, in practice, a large knowledge gap in identifying or diagnosing myoclonus [13]. Indeed, estimates of the socioeconomic burden of all-cause myoclonus are scarce, but a recent study suggests a significant contribution of drug-induced myoclonus to increased healthcare costs due to patient-reported poor quality-of-life, healthcare utilization, and the need for prompt management [14].

It is likely that outpatient and inpatient presentations of myoclonus differ in terms of symptom duration and overall presentation. For example, our prior study noted that most inpatient cases of myoclonus resulted from toxic-metabolic and hypoxic-ischemic etiologies [13]. A population study out of Olmstead County, Minnesota by Caviness *et al*. (1999) found symptomatic myoclonus to be most prevalent (72%), followed by epileptic (17%) and essential (11%) myoclonus [15]. Our current study seeks to compare the demographic and etiological characteristics of inpatient and outpatient individuals with myoclonus from a single large urban center.

## Methods

### Study Design and Subject Enrollment

The New York Presbyterian Brooklyn Methodist Hospital Institutional Review Committee approved this retrospective chart review with a waiver of informed consent due to anonymization of patient data and the impracticality of obtaining informed consent from a large sample (IRB# 1712095). For this retrospective cohort study, patients whose diagnoses included ICD-10-CM codes for “myoclonus” and “asterixis” (G25.3) from 2011 to 2021 (a 10-year period) were compiled on the Electronic Medical Record for New York Presbyterian Brooklyn Methodist Hospital (Brooklyn, New York, United States of America) into an Institutional Review Board approved database that was de-identified for statistical analysis after data collection was completed. Codes for other conditions such as myoclonic epilepsies were not explicitly searched for as these conditions presented primarily in the pediatric population, who were excluded from this study.

Subjects were included if they were adult individuals (21 years of age or older) seen by a neurologist in the outpatient clinical setting from 2010 to 2020 with explicitly documented myoclonus or asterixis in the objective clinical examination and impression/recommendation sections of consultation and/or progress notes. The following were excluded from the study: pediatric patients (younger than 21 years of age); patients with alternative diagnoses (that is, movement disorders other than frank myoclonus); patients without explicitly documented myoclonus in the charts’ physical examination, assessment, and recommendation sections; patients who were not seen by a neurologist; and patients with limited clinical information. Although descriptors for myoclonus such as “jerk” or “twitch” were encountered in the history sections of consultation notes, these descriptors were not utilized as inclusion criteria unless the other sections explicitly stated “myoclonus” or “asterixis”.

The records were abstracted for data at the time by five medical residents (D.Z., M.O., A.Z, J.E., and S.R.), a fourth-year medical student (K.O.), and one movement disorder neurologist (M.H.). The movement disorder neurologists (M.H., H.O., H.S., D.V., and M.S.) reviewed all records for standardization of abstracted clinical information and satisfaction of inclusion/exclusion criteria.

### Study Procedures

Included charts were then searched extensively for extractable data, including demographic data (age at presentation, gender, and ethnicity); duration of symptoms before presentation to the outpatient setting; myoclonus semiology (location and provoking maneuvers); myoclonus etiology (idiopathic, physiological, spinal nervous system lesions, toxic/drug-induced [previously iatrogenic], metabolic [non-genetic], static lesions causing seizures, static hypoxic-ischemic nervous system lesions, inflammatory/autoimmune/paraneoplastic, progressive neoplastic lesions, neurodegenerative, and functional); and treatment response and outcomes. Treatment response was defined as subjective and objective changes in myoclonus as defined by patient history and physician examination, usually at subsequent follow-up.

### Statistical Analysis

Descriptive statistics were summarized as frequency with percentages, mean, median, and range used for categorical and continuous factors, respectively. The Chi-square test or Fisher’s exact test was used, as appropriate, to compare the proportions between variables of interests (gender, ethnicity, race, etiology, myoclonus semiology, etc.) and outcome (mortality) in both settings. The relationship between the duration of symptoms or age and other variables were analyzed using Wilcoxon rank-sum test. All analyses were performed in SAS Version 9.4 (SAS Institute, Inc., Cary, NC).

## Results

### Demographics

**Figure 1:**
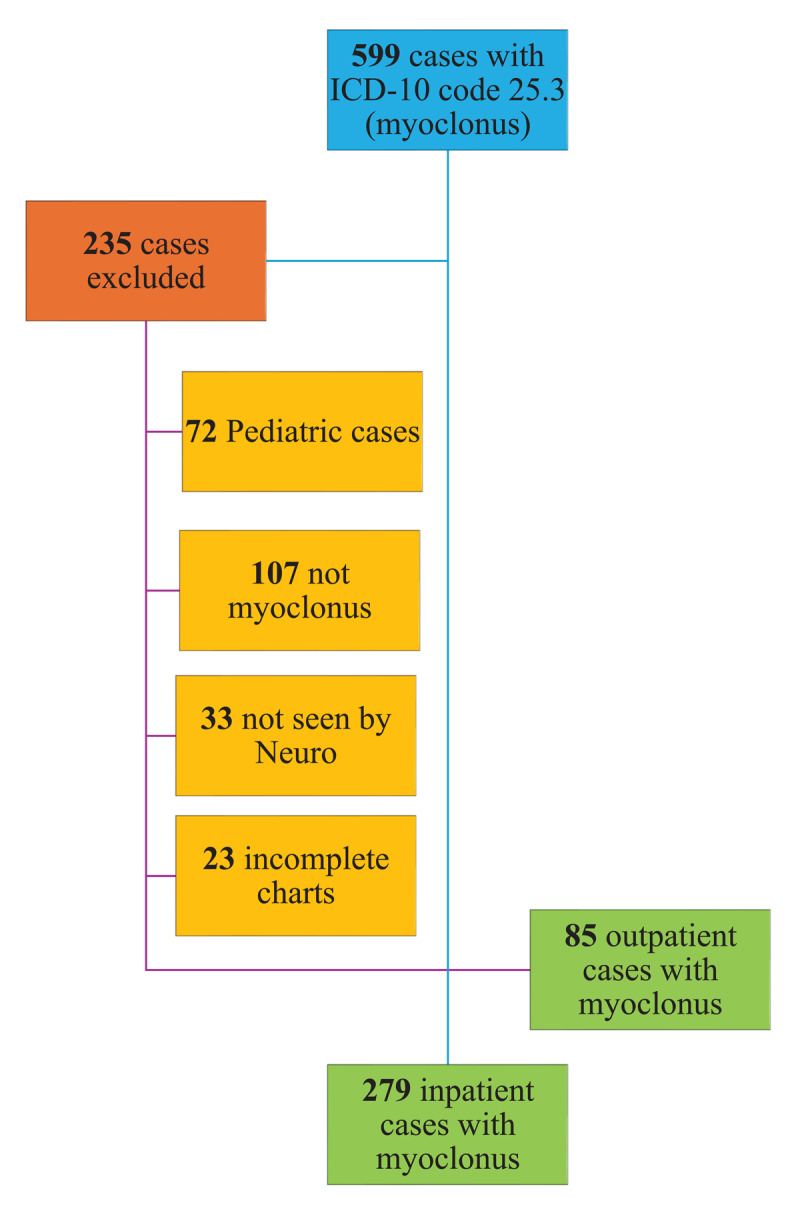
CONSORT (Consolidated Standards of Reporting Trials) flowchart detailing study sample size and exclusion criteria.

Two-hundred seventy-nine individuals, 56.63% female, met the inclusion criteria for our inpatient cohort, aging at 70.61 + 15.76 years [13]. Eighty-five individuals met the inclusion criteria and were included in our outpatient cohort (Figure 1). A total of 235 charts were excluded from the study: 107 had diagnoses apart from frank myoclonus or asterixis, 72 were pediatric cases, 33 were not seen by a neurologist, and 23 had insufficient chart data available (Figure 1).Of those included, 52.94% were female and aged 64.3 + 16.3 years. The outpatient group had a younger median age at evaluation of 66 years compared to our inpatient cohort (74 years), with the difference in median age between the two populations being statistically significant (*p* = 0.001). Most outpatient cases were Caucasian (42.35%) or black (32.94%) compared to 45.52% black and 32.97% Caucasian in the inpatient cohort, but the discrepancy in ethnic distributions was not statistically significant on Chi-square testing (*p* > 0.05). The duration of symptoms in the outpatient setting prior to presentation, with a median of 15 months (IQR 3–45 months), was longer than in the inpatient setting, with a median of less than a day (IQR < 1–4 days); the difference was statistically significant (*p* < 0.001). Of all outpatient cases, 67.1% were seen by a Movement Disorders specialist, a significantly higher proportion compared to 44.8% of cases in the inpatient setting (*p* < 0.001).

### Etiological Breakdown

**Figure 2:**
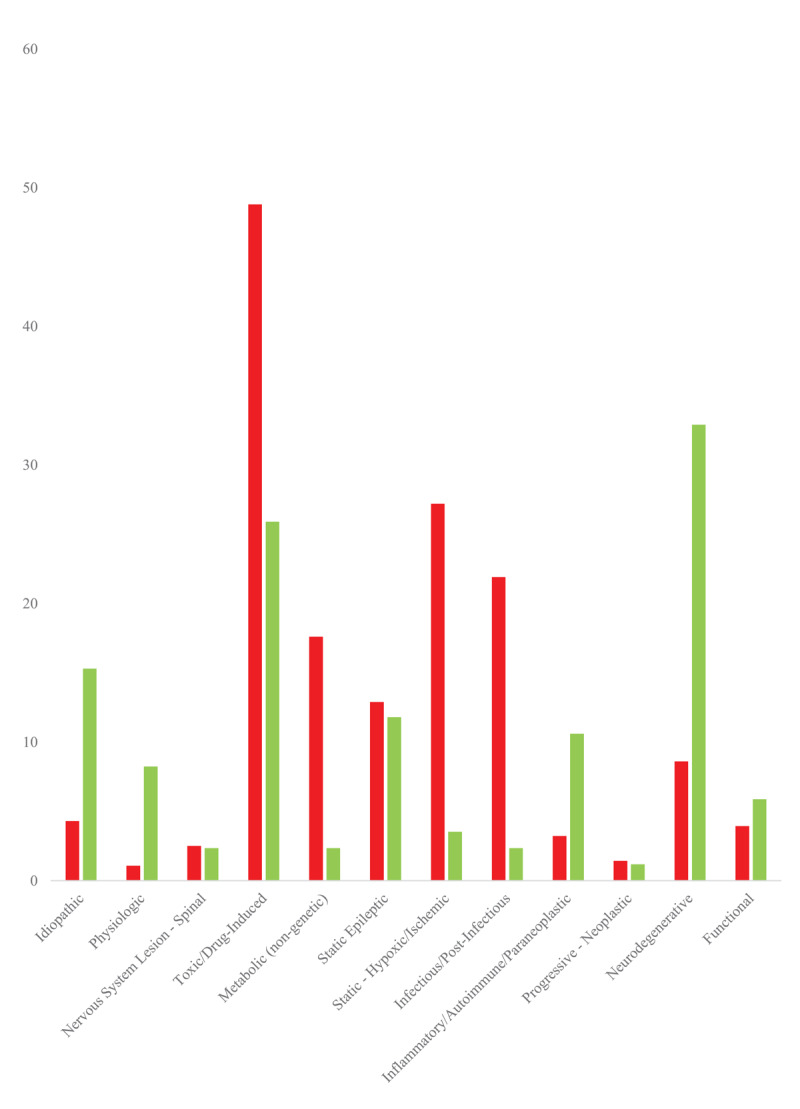
Bar graph detailing number of cases per identified etiology for myoclonus in the inpatient (red) and outpatient (green) settings. The total number of inpatient charts reviewed was 279 individuals, whereas the total number of outpatient charts reviewed was 85 individuals. Almost half of studied cases in the inpatient setting had one probable etiology, whereas the remainder had 2 or more probable etiologies. More than half of the outpatient cases exhibited a singular probable etiology.

Most outpatient cases had only 1 etiology (71.8%) while the remaining had 2 or more etiologies. This contrasts with the inpatient population, where the division between having 1 etiology versus 2 or more etiologies was more evenly split (that is, approximately 50% of inpatient individuals had a single etiology whereas the remaining 50% had 2 or more probable etiologies). The difference in number of concomitant etiologies between both settings was statistically significant on Fisher’s exact test (*p* < 0.05). While most cases encountered in the outpatient setting were symptomatic due to underlying neurological and/or systemic illnesses, 15.3% and 8.24% of individuals had idiopathic and physiologic myoclonus respectively, both substantially larger (*p* < 0.05) than in our previously reported inpatient cohort (4.30% and 1.08% respectively). Additionally, idiopathic myoclonus demonstrated a difference in odds ratio, albeit not a statistically significant one (*p* > 0.05), between individuals younger than 75 years of age (0.34) and those older than 75 years of age (0.87). Most symptomatic cases in the outpatient cohort were a result of neurodegenerative (32.9%), toxic/drug-induced (25.9%, most commonly from gabapentin and/or sertraline), and autoimmune/paraneoplastic (10.6%) etiologies. These proportions differed significantly (*p* < 0.05) from the inpatient cohort, who exhibited primarily toxic-metabolic (both toxic/drug-induced and metabolic/non-genetic, 48.8% and 17.6% respectively for inpatient versus 25.9% and 2.35% % for outpatient respectively), and static hypoxic-ischemic (27.2% versus 3.53%). All these differences were statistically significant (*p* < 0.05). Less common etiologies, namely static epileptic, spinal nervous system lesion, progressive neoplastic, and functional myoclonus, were similar in proportions between the two groups (*p* > 0.05). These differences can be seen in Figure 2. Further etiological breakdowns of cases encountered are tabulated in Table 2.

**Table 2:** Sample of final diagnoses encountered per class of etiology in inpatient and outpatient settings incorporating the IAPRD 2025 consensus classification.


ETIOLOGY	DIAGNOSES

**Metabolic (non-genetic)**	Hepatic encephalopathy Uremic encephalopathy Hyponatremia Hyperglycemic non-ketotic state Diabetic ketoacidosis Dialysis disequilibrium syndrome

**Toxic/drug-induced (iatrogenic)**	Gabapentin-induced myoclonus Sertraline toxicity Budesonide/formoterol toxicity Quetiapine toxicity Phenytoin-induced myoclonus Tramadol-induced myoclonus Opiate/narcotic withdrawal Alcohol withdrawal

**Nervous system lesions (cerebral hypoxic-ischemic)**	Cerebrovascular disease (post-stroke) Lance-Adams myoclonus Anoxic brain injury Intraparenchymal hemorrhage Hypoxic-ischemic encephalopathy

**Nervous system lesions (spinal)**	Cervical spinal stenosis Propriospinal myoclonus

**Progressive (neoplastic)**	Cerebral meningioma Intracranial metastases T-Cell lymphoma

**Neurodegenerative disease**	Parkinson’s Disease Multiple System Atrophy Dementia with Lewy Bodies Corticobasal ganglionic degeneration Frontotemporal dementia Tuberous sclerosis Essential tremor with myoclonus

**Infectious/post-infectious**	Urinary tract infection Septic encephalopathy COVID-19 pneumonia Creutzfeldt-Jakub Disease Transverse myelitis

**Epileptic (static)**	Juvenile-onset myoclonic epilepsy Myoclonic status epilepticus Symptomatic epilepsy with myoclonus

**Functional**	Psychogenic non-epileptic spells (PNES) Functional myoclonus-like movement disorder

**Physiologic**	Singultus (hiccups) Hypnagogic myoclonus

**Idiopathic**	Essential myoclonus


No statistically significant gender or racial differences were noted by etiology. Neurodegenerative etiologies were more prevalent in those over age 75 years at presentation in our outpatient cohort (*p* < 0.05), but this age preference was not seen in neurodegenerative cases encountered in the inpatient setting (*p* > 0.05). In terms of semiology, spinal nervous system lesion (i.e. originating from a spinal cord lesion) and toxic/drug-induced myoclonus cases, particularly in the outpatient group, were more associated with focal myoclonus (12.5% and 37.5% respectively), involving more axial (truncal, facial and bulbar muscles) than appendicular (upper and/or lower extremity) musculature (*p* < 0.05). Idiopathic and static epileptic myoclonus were more associated with appendicular (upper and lower extremity) involvement (30% and 16.7% respectively), particularly in the inpatient group (*p* < 0.05). Despite this, all outpatient cases were predominantly appendicular (over 80% of cases) in semiology, whereas all inpatient cases were predominantly generalized (approximately 70% of all cases). Only two individual cases in the outpatient cohort involved asterixis, both occurring from chronic renal disease and no outpatient data were available for stimulus-sensitive or action-induced cases of myoclonus. In contrast, 26 (12.2%) of inpatient cases exhibited asterixis, with 92.3% associated with metabolic non-genetic etiologies (80.8% renal), and 20 (9.4%) of inpatient cases involved stimulus-sensitive myoclonus, with 65% of those cases being static hypoxic-ischemic in etiology.

### Treatment Outcomes

Treatment of underlying associated etiologies was given to 68.2% of our outpatient cohort (whereas the rest received symptomatic treatment), with 75.9% of these individuals reporting good response or physical improvement in myoclonus noted by treating physician at follow-up. A similar etiologic treatment response rate of 70% was seen in our inpatient cohort, with metabolic non-genetic and toxic/drug-induced etiologies exhibiting the best response rates (i.e. nearly 100% of the time), and hypoxic-ischemic etiology showing only less than 40% response to etiological treatment (supportive care).

Symptomatic treatment was also analyzed. Levetiracetam was the most used anti-epileptic drug to treat myoclonus in our outpatient cohort, with 20 of 26 (76.9%) individual cases showing good response to treatment, defined as improvement in myoclonus at follow-up as per treating physician. This was followed by clonazepam (9 of 16, 56.3%) and other anti-epileptic drugs (14 out of 21, 66.7%) such as valproic acid. A similar response rate to levetiracetam (75.8%) was seen in our inpatient cohort. Additional data was available for 2^nd^ and 3^rd^ anti-seizure drug use in the inpatient cohort, with diminishing returns in terms of treatment response (48% and 39%) compared to those who were on one ASD (76%), with a higher mortality rate in non-responders to a third agent (66.7%). The differences in these proportions were statistically significant.

Finally, our inpatient cohort exhibited an overall 27.2% mortality rate, mostly associated with static hypoxic-ischemic etiologies (*p* < 0.001) and least associated with neurodegenerative and idiopathic etiologies (*p* < 0.05). No reliable mortality data was available for the outpatient cohort as many were lost to subsequent follow-up.

## Discussion

The primary objectives of this study are to elucidate the distribution of demographics, etiology, semiology, and treatment outcomes of individuals with myoclonus in the outpatient setting and compare – where available and applicable – these parameters to our previously described inpatient cohort. We found that symptomatic myoclonus was more prevalent in both of our inpatient and outpatient cohorts compared to idiopathic and functional myoclonus, although idiopathic myoclonus was much more common in the outpatient setting than the inpatient setting (15.3% versus 4.30%). Of the symptomatic outpatient cases, most were due to neurodegenerative, toxic/drug-induced, or inflammatory/autoimmune/paraneoplastic causes, whereas symptomatic inpatient cases were predominantly due to toxic/drug-induced, metabolic (non-genetic), or static hypoxic-ischemic etiology. Our outpatient population was also younger on average, predominantly Caucasian, and more likely exhibited a single etiology compared to the inpatient population. Spinal nervous system lesion and toxic/drug-induced cases, particularly in the outpatient cohort, were the most focal in their semiology and involved more axial than appendicular musculature. Generalized and multifocal semiologies are more associated with metabolic (non-genetic) and neurodegenerative etiologies. Treatment response was present in many of the outpatient cohort cases who had follow-up. Etiology-specific response rate was unable to be done reliably for the outpatient cohort, likely owing to the smaller sample size in our cohort.

While our study did not investigate myoclonus on an epidemiological basis, at least one previous study elucidated the incidence and prevalence of myoclonus in general populations. Caviness *et al*. (1999) looked at the incidence and prevalence of myoclonus in Olmsted County, Minnesota, from 1976 to 1990 and found that myoclonus cases increased particularly in males with advancing age [15]. In the same study, cases of symptomatic myoclonus (72%), namely those associated with neurodegenerative conditions such as progressive supranuclear palsy, Alzheimer’s disease, and Creutzfeldt-Jakub disease, were the most prevalent, followed by epileptic myoclonus (17%) and essential, or primary/idiopathic, myoclonus (11%) [15]. Our study disclosed a relatively equal proportion of males and females without any statistically significant difference in age between the two subgroups (*p* > 0.05 on Student two-tailed *t*-test assuming equal variances). Moreover, the proportion of idiopathic myoclonus in our study, 15.3%, was higher than that previously reported in the Olmsted County census. Caviness *et al*. postulated that Olmsted County’s population was more ethnically homogenous, and familial cases of hereditary essential myoclonus aggregated in this area [15].

Provoking movements of and semiologies for myoclonus have been investigated in neurological and medical literature. Generalized myoclonus, historically associated with toxic/drug-induced, metabolic (non-genetic), and neurodegenerative etiologies, strongly associated predominantly with the same etiologies in our inpatient cohort, whereas focal myoclonus preferentially associated with static epileptic etiologies in our inpatient cohort [13]. These associations were not statistically significant in our outpatient cohort, likely owing to sample size limitations (*p* > 0.05). Previous studies noted an abundance of asterixis, so-called “negative myoclonus”, in the inpatient setting compared to the outpatient setting, most likely owing to higher incidence and/or prevalence of metabolic (non-genetic) disease in the acute setting [13,16,17,18]. Indeed, our study only found 2 out of 85 cases (2.4%) with asterixis in the outpatient setting, both arising from chronic kidney disease, and this is consistent with findings in other studies [17]. In contrast, less than 17.6% of 279 inpatient cases exhibited underlying renal etiology and comprised 90% of all inpatient cases in our cohort with asterixis [13]. The severity of asterixis, while not investigated here, has been correlated in literature with increasing renal dysfunction as evidenced by rising blood urea nitrogen and creatinine parameters [18]. More interesting are our findings regarding spinal nervous system lesion myoclonus in our outpatient cohort: more axial than appendicular features were present. While not completely novel, this finding supports prior research in individuals with axial myoclonus of myelopathic origin [19,20,21]. Historically, both spinal myoclonus and propriospinal myoclonus more commonly involve axial jerks and twitches that may change with movement of the spinal column such as when laying supine or standing for prolonged periods [19,20,21].

Finally, treatments for myoclonus vary depending on the underlying etiology, but additional symptomatic treatment is reserved for those individuals where myoclonus becomes disabling or even dangerous when carrying out activities of daily living, such as feeding and grooming. Levetiracetam is one of the most commonly used treatments, along with clonazepam and valproic acid [22]. However, most likely due to its favorable side effect profile relative to the other two anti-seizure drugs, levetiracetam use was prevalent in our cohort with an excellent response rate as evidenced by improvement in myoclonus at follow-up assessments by the treating physicians. Unfortunately, owing to small sample size and significant loss to follow-up, no direct comparisons of treatment response and outcomes such as mortality could be made between our inpatient and outpatient cohorts.

As in our previous inpatient study, our outpatient comparison study is marred by several limitations, most significantly being sample size and selection bias. The Electronic Medical Record’s search engine procured 599 charts for the study: 279 for inpatient myoclonus and only 85 for outpatient myoclonus. This difference was somewhat ameliorated with chart abstraction oversight by neurologists trained in movement disorders, and the fact that 67.1% of outpatient cases were seen by a movement disorders specialist. This widens the uncertainty regarding any conclusions of differences between the cohorts. Second, no long-term data was available due to significant loss to follow-up, and long-term outcomes could thus not be assessed for most cases in the outpatient cohort. Despite these limitations, most of the patients had a single explanatory etiology, and conclusions about etiology-specific characterizations are more robust in this study than they were in our inpatient study. Additionally, no genetic analyses have been performed on our patients, and genetic etiologies were thus not fully captured in our sample. The same can be said for electrophysiological data, as limited resources prevented large-scale utility of both electroencephalography and electromyography in the inpatient and outpatient settings. Multiple etiologies and lack of electrophysiological data limit our ability to determine the drivers or generators of myoclonus in a substantial proportion of our patients. Finally, our charts lacked scores for examining myoclonus such as the Unified Myoclonus Rating Scale, and the absence of uniform clinical myoclonus evaluations across charts thus limit conclusions on localization; however, prior literature in this field has made use of subjective and objective clinical examinations as evidence for treatment response, and this is a prevalent issue that has been described elsewhere in literature [23]. Therefore, the rate of success of symptomatic treatments as reported in this study could not be reflective of the anticipated response if treatments were to be given according to the type of myoclonus, even if treatment was based on Axis 1a localizations only.

In conclusion, and supportive of prior research in this area, etiology of myoclonus differs between the inpatient and outpatient settings. Inferences about localization and etiology of myoclonus can be made with history-taking and careful clinical examination, although a great degree of variability in presentation exists even within etiological subgroups (Table 2). Treatments should be focused on symptomatic and pathophysiological paradigms. Future research with analysis of prospective clinical and electrophysiological data, with consideration of etiological and therapeutic data, is needed to address knowledge gaps in myoclonus symptomatology and treatment response.

## Data Accessibility Statement

De-identified data that support the findings of this study are available from the corresponding author, MH, upon reasonable request.
